# Gut carriage of antimicrobial resistance genes among young children in urban Maputo, Mozambique: Associations with enteric pathogen carriage and environmental risk factors

**DOI:** 10.1371/journal.pone.0225464

**Published:** 2019-11-22

**Authors:** David Berendes, Jackie Knee, Trent Sumner, Drew Capone, Amanda Lai, Anna Wood, Siddhartha Patel, Rassul Nalá, Oliver Cumming, Joe Brown

**Affiliations:** 1 Division of Foodborne, Waterborne, and Environmental Diseases, U.S. Centers for Disease Control and Prevention, Atlanta, Georgia, United States of America; 2 School of Civil and Environmental Engineering, Georgia Institute of Technology, Atlanta, Georgia, United States of America; 3 Department of Epidemiology, Rollins School of Public Health, Emory University, Atlanta, Georgia; 4 National Institute of Health, Maputo, Mozambique; 5 Department of Disease Control, London School of Tropical Medicine and Hygiene, London, United Kingdom; Purdue University, UNITED STATES

## Abstract

Because poor sanitation is hypothesized as a major direct and indirect pathway of exposure to antimicrobial resistance genes (ARGs), we sought to determine a) the prevalence of and b) environmental risk factors for gut carriage of key ARGs in a pediatric cohort at high risk of enteric infections due to poor water, sanitation, and hygiene (WASH) conditions. We investigated ARGs in stool from young children in crowded, low-income settlements of Maputo, Mozambique, and explored potential associations with concurrent enteric pathogen carriage, diarrhea, and environmental risk factors, including WASH. We collected stool from 120 children <14 months old and tested specimens via quantal, multiplex molecular assays for common bacterial, viral, and protozoan enteric pathogens and 84 ARGs encoding potential resistance to 7 antibiotic classes. We estimated associations between ARG detection (number and diversity detected) and concurrently-measured enteric pathogen carriage, recently-reported diarrhea, and risk factors in the child’s living environment. The most commonly-detected ARGs encoded resistance to macrolides, lincosamides, and streptogramins (100% of children); tetracyclines (98%); β-lactams (94%), aminoglycosides (84%); fluoroquinolones (48%); and vancomycin (38%). Neither concurrent diarrhea nor measured environmental (including WASH) conditions were associated with ARG detection in adjusted models. Enteric pathogen carriage and ARG detection were associated: on average, 18% more ARGs were detected in stool from children carrying bacterial pathogens than those without (adjusted risk ratio (RR): 1.18, 95% confidence interval (CI): 1.02, 1.37), with 16% fewer ARGs detected in children carrying parasitic pathogens (protozoans, adjusted RR: 0.84, 95% CI: 0.71, 0.99). We observed gut ARGs conferring potential resistance to a range of antibiotics in this at-risk cohort that had high rates of enteric infection, even among children <14 months-old. Gut ARGs did not appear closely correlated with WASH, though environmental conditions were generally poor. ARG carriage may be associated with concurrent carriage of bacterial enteric pathogens, suggesting indirect linkages to WASH that merit further investigation.

## Introduction

Antimicrobial resistance (AR) is a major global health threat, with antimicrobial use and resistance concurrently growing worldwide. From 2000–2010, antimicrobial use increased by >30% globally, with the largest increases in low- and middle-income countries (LMICs), likely due to the high burden of infectious diseases and the increased availability and reduced cost of antimicrobials [1]. A recent birth cohort study of seven LMIC sites found 98% of infants were exposed to antimicrobials by 6 months of age [2]. Antimicrobial use in LMICs may be high due to both insufficient training or oversight into prescriber practices, ease of access and lack of regulation of antimicrobial agents to avoid use outside of pharmaceutical and clinical settings, and mass drug campaigns that include administration of antimicrobials to healthy children [1,3,4]. Thus, selective pressure from human use of antimicrobials in LMICs is increasing. However, evaluation and assessment of AR, and risk factors for antimicrobial-resistant infections, in LMICs remains limited because of poor surveillance and insufficient funding [1].

Emerging regional- and national-scale evidence suggests poor water, sanitation, and hygiene (WASH)—directly and indirectly—facilitates the development and environmental dispersion of AR organisms [5–8]. More directly, poor WASH conditions—especially poor containment and treatment of AR organisms and their genes through the sanitation chain—may facilitate the development of resistance and increase individual risks of exposure to AR genes (ARGs) or AR (vs. susceptible) pathogens [6,7,9,10]. Indirectly, poor WASH conditions result in children exposed to enteric pathogens more frequently and in potentially higher concentrations [11,12], increasing treatment with antibiotics or antimicrobials and thereby accelerating the development of AR [10].

However, the hypothesized environmental contributions to ARG exposures have not been directly measured on local scales, especially in LMICs where poor WASH infrastructure may be highly prevalent and of paramount concern [10,13]. Insufficient treatment of AR organisms and ARGs at sewage or fecal sludge treatment plants, and persistence of antibiotics in the fecal waste stream, put water sources and water-associated environmental transmission pathways at particular risk of contamination [6,14–18]. Furthermore, many LMIC communities lack consistent access to functional, safe management of human waste throughout the sanitation chain, leading to environmental discharge of untreated wastes [19–21]. More than half of the world’s population and their feces (and almost two-thirds in LMICs [19]) are managed in onsite systems [22] and 60% of the world uses unsafely-managed sanitation [20], bringing exposure risks directly into the household or the peripheral environment. Carriage of bacterial enteric pathogens, due to exposure to poor WASH conditions [11], may co-occur with AR either because enteric bacteria carry ARGs or because ARGs may accompany diverse bacterial exposures.

Methodologically, challenges exist to assessing the fate, transport, and burden of AR from the environment to humans and its impact on human health. Culture-based methods remain the standard for assessing phenotypic resistance of AR organisms in the environmental and in the gut microbiome, and are widely used for surveillance and health impact assessments [13,23]. Scaling these methods to large studies is constrained by human and financial resources [13]. Conversely, molecular approaches to ARG detection present the potential for high-throughput screening. However, interpretation of ARG carriage is challenging, given limited understanding of their background circulation and clinical implications without culture confirmation [23,24]. Molecular approaches are moving towards more accurate prediction of phenotypic resistance [25]. Additionally, there is a need to understand concurrent ARG prevalence in environmental media like sewage, wastewater, soil, drinking water, and recreational water, particularly in environments with high fecal contamination [9]. As a start, an in-depth understanding of the background prevalence and risk factors for ARGs in human hosts in these settings can characterize the epidemiology and potential for environmental measures to prevent ARG transmission.

In order to evaluate direct and indirect links between poor WASH (environmental risk factors) and ARG transmission, we evaluated ARG prevalence and associations between total number of ARGs detected (and diversity of ARGs detected) and 1) environmental risk factors (direct links: e.g. poor sanitation); and 2) enteric pathogen carriage (indirect links), in children born into a pathogen-rich, densely-populated urban environment in Maputo, Mozambique as part of a larger health impact trial of sanitation [26]. The goals of this study were to: a) evaluate the prevalence and diversity of ARGs in young children (<14 months old) in a low-income, urban environment; b) assess risk factors for ARGs in these children; and c) compare ARG outcome metrics of diversity and total genes from a qPCR array including pre-selected ARGs. Use of a qPCR array with pre-selected ARGs enabled comparison of relative AR profiles between children in varying environments. Results from this analysis can improve understanding of background circulation of ARGs in children in Mozambique and settings with similarly poor infrastructure and high burdens of enteric infections, and of links between environmentally-mediated risk factors and ARGs.

## Methods

This study was nested within a subgroup of children enrolled from 2015–2017 in the Maputo Sanitation (MapSan) trial in Maputo, Mozambique [26]. Briefly, the MapSan trial tested the effects of a shared private sanitation intervention–pour-flush latrines with septic tanks and soakaway pits–on child health as compared to existing poor sanitation. In this setting, sanitation is shared at the compound level; compounds are groups of two or more houses that share outdoor living space (and sanitation). Thus, this study was a sub-analysis that the original MapSan study was not designed to explicitly evaluate. For this study, the first 60 stools (one per child) analyzed for the MapSan study that were from children <14 months old enrolled in MapSan control and intervention compounds in round 1 (February 2015 –February 2016, pre-intervention) and the first 60 meeting the same criteria (that is, among new children born into an intervention or control compound) in round 2 (March 2016-April 2017, 12 months post-intervention) were selected (n = 120 stools total). Selection was purposefully split evenly by group: 30 children per intervention or control group in each round. Enrollment age criteria ensured children were born into compounds following any changes in sanitation conditions associated with the intervention (if in that group). By design of this study and enrollment age criteria, no children could be enrolled multiple times, but a compound could be enrolled multiple times. Statistical models addressed potential within-compound level of clustering using random effects for the compound of the child. Enteric pathogens in stool specimens were assessed by a multiplex RT-PCR assay (Luminex xTAG Gastrointestinal Pathogen Panel (GPP)^®^, Luminex Corp, Austin, TX, USA). The GPP tests for 15 enteric pathogens: 9 bacteria (*Campylobacter*, *C*. *difficile*, enterotoxigenic *E*. *coli* (ETEC), Shiga toxin-producing *E*. *coli* (STEC), *E*. *coli* O157, *Salmonella*, *Shigella*, *V*. *cholerae*, *Y*. *enterocolitica*), 3 viruses (adenovirus 40/41, norovirus GI/GII, rotavirus A), and 3 amoeba/protozoa (‘parasites’: *Giardia*, *Cryptosporidium*, *Entamoeba histolytica*). The GPP is a well-validated assay with high sensitivity and specificity that has been tested across multiple countries [27–33]. Child stool specimens were also assessed for 84 ARGs via a commercial qPCR ARG array (the Antibiotic Resistance Genes Microbial DNA qPCR array, Qiagen, Valencia, CA, USA, ARGs in S1 Table), which has been used previously to characterize ARGs in the human gut, environmental soil, and meat for consumption in high-income settings [34–38]. Ethical approvals for stool and survey data collection were obtained from the Comité Nacional de Bioética para a Saúde, Ministério da Saúde (333/CNBS/14), the Ethics Committee of the London School of Tropical Medicine and Hygiene (reference # 8345), and the Institutional Review Board of the Georgia Institute of Technology (protocol # H15160). Ethical committees approved the verbal consent procedures because most respondent were illiterate. All consents were recorded on the survey questionnaire or tablet prior to initiating surveys. The MapSan trial was registered at ClinicalTrials.gov (NCT02362932).

### Study site

The study site comprised densely-populated, low-income neighborhoods of Maputo, Mozambique described elsewhere [26,39]. Briefly, children lived in households organized in compounds that generally shared a sanitation facility. Most households had access to a sanitation facility that contained waste onsite, while few lacked access to basic sanitation facilities [39]. Children from these compounds had to be ≥1 month old to be enrolled in MapSan [39].

### Surveys and observation

Data were collected on child, household, and compound demographics; water, sanitation, and hygiene (WASH) practices; and household wealth by surveys and observation [39]. The child’s mother was the target respondent for surveys about the child’s health and the household. If the mother was unavailable, another parent or guardian was asked to respond to survey questions. The head of compound, or their spouse, was the target respondent for surveys about the compound itself. Observations of household conditions, WASH infrastructure, and the compound overall were also completed. Household wealth was measured by an asset-based index specific to and locally-validated in Mozambique [40].

### Stool collection and analysis

Stool specimens were collected as described previously [39]. Briefly, caregivers were given diapers or containers for collecting children’s stool with pre-labelled sample bags and to store collected stool in a cool, dry place in the household. Stool specimens were picked up from the household the following day, and transported on ice to the Mozambican Ministry of Health (MISAU/INS) within 6 hours for storage at -80°C. Specimens were then shipped on dry ice, with temperatures monitored, to the Georgia Institute of Technology for storage at -80°C until analysis.

Stool specimens were analyzed by the GPP according to manufacturer’s instructions, using the QIAcube HT platform and QIAamp 96 Virus QIAcube HT Kit (Qiagen, Hilden, Germany). Extracted nucleic acids were stored at 4°C and analyzed by GPP within 24 hours. Further details on stool analysis for enteric pathogens can be found in Knee et al. [39].

For ARG assessment, DNA from whole stool was extracted using the Qiagen PowerFecal Kit for Qiacube (Qiagen, Hilden, Germany). Extracted DNA was stored at -80°C until use, and underwent ≤1 previous freeze-thaw cycle before analysis. Total reaction volumes, including amount of Qiagen Microbial DNA Mastermix and target amount of DNA (500ng/array) were set according to manufacturer’s instructions. Arrays were loaded and run on an ABI 7500 Real-Time PCR System (Applied Biosystems, Foster City, CA, USA) following manufacturer’s instructions or stored at -20°C for up to 7 days until the run. Quantification cycle (C_q_) values of <34 indicated positive detects. Each sample had six pan-bacteria positive controls (to ensure sufficient quantities of bacterial DNA) and three plate controls (tests for inhibitors via artificial sequence and primers). Lower limits of quantification for most ARG targets in the assay (97%) were <100 gene copies per reaction.

### Aggregate ARG indices

Beyond individual ARG prevalence, ARGs detected in each child’s stool specimen were evaluated by three aggregate measures: total ARGs, Shannon Index, and Inverse Simpson’s Index. Total ARGs were calculated as the total number of positive wells (targets) detected per specimen. In ecological literature, diversity indices (Shannon Index [41] and Inverse Simpson’s Index [42]) measure the richness of individual species (generally: [number of species represented]:[number of individuals in each species], thus higher numbers indicate greater diversity) and each give different weights to dominant species. They have been used to quantify the diversity of mobile genetic elements and ARGs in infants in high-income settings [43] and of ARGs in environmental media (e.g. sewage sludge [44,45]). In this study, ARG groups (e.g. aminoglycoside resistance, tetracycline resistance; β-lactamase resistance subdivided into Ambler classes (class A-D [46])) represented the ‘species’ level, with number of targets detected within each ARG group representing the ‘number of members of the species’ in ecological terms.

### Statistical analyses

All data were analyzed in R version 3.4.0 (R Foundation for Statistical Computing, Vienna, Austria [47]) using the ‘lme4’ package for generalized linear models with random effects [48] and the ‘vegan’ package for calculating Shannon and Inverse Simpson’s indices [49]. The ‘total ARG’ outcome, the number of ARGs detected, was analyzed using mixed-effects Poisson regression, with a random effect for the child’s compound. Diversity indices were analyzed using mixed-effects linear regression, with a random effect for the compound. Demographic, WASH, and wealth risk factors that were tested for associations with these outcomes were included as continuous or categorical variables. For categorical variables with >2 levels, dummy variables were created: e.g. for sanitation, children in compounds with a pit latrine were the referent group, compared with children in compounds with pour flush facilities compared to them. All analyses were adjusted for round of enrollment. Enteric pathogen risk factors (enteric pathogens detected in stool) were also adjusted for reported diarrhea to encompass symptomatic enteric infections that may cause caregivers to seek care for the child with antibiotics [2].

## Results

### Demographic characteristics and enteric infections

We assessed demographic characteristics and enteric pathogen carriage in study children by surveys and stool specimen analysis, respectively (Table 1). All children were <14 months old (average age of 8 months, standard deviation (SD): 3 months; Table 1a) and half (53%) were female. Most children (80%) were breastfed at the time of survey, but only 17% of those breastfed were exclusively breastfed. Most households had access to a pit latrine (74%), and mean value for the (unitless, ranging from 0–1) wealth index was 0.39.

**Table 1:** Demographic characteristics and enteric infections among children < 14 months old, Maputo, Mozambique^1^.

a) Demographics	Mean (standard deviation (SD)) or N (%), Round 1	Mean (SD) or N (%), Round 2	Mean (SD) or N (%), all years
Age (months)	7.5 (3.2)	8.7 (2.8)	8.1 (3.0)
Female child^2^	33 (56%)	30 (50%)	63 (53%)
Child is breastfed	46 (77%)	50 (83%)	96 (80%)
Child is exclusively breastfed	12 (26%)	4 (8%)	16 (17%)
Wealth index (unitless, range: 0–1)	0.43 (0.13)	0.35 (0.14)	0.39 (0.14)
Type of toilet			
Pit latrine	59 (98%)	30 (50%)	89 (74%)
Pour flush to septic tank	0	27 (90%)	27 (23%)
Pour flush to other	1 (2%)	3 (10%)	4 (3.3%)
b) Reported diarrhea			
Diarrhea in last 7 days^3^	9 (18%)	8 (18%)	17 (18%)
Sought treatment for diarrhea	6 (67%)	3 (38%)	9 (53%)
c) Enteric pathogen detection (stool specimens)			
Any pathogen	51 (85%)	48 (80%)	99 (83%)
Number of pathogens			
0	9 (15%)	12 (20%)	21 (18%)
1	27 (45%)	20 (33%)	47 (39%)
2	15 (25%)	23 (38%)	38 (32%)
3	8 (13%)	4 (7%)	12 (10%)
4	0	0	0
5	1 (2%)	1 (2%)	2 (2%)
Bacterial pathogens^4^	43 (72%)	40 (67%)	83 (69%)
Number of bacterial pathogens			
0	17 (28%)	20 (33%)	37 (31%)
1	29 (48%)	23 (38%)	52 (43%)
2	13 (22%)	15 (25%)	28 (23%)
3	1 (2%)	1 (2%)	2 (2%)
4	0	1 (2%)	1 (1%)
Parasitic pathogens^5^	13 (22%)	10 (17%)	23 (19%)
Number of parasitic pathogens			
0	47 (78%)	50 (83%)	97 (81%)
1	11 (18%)	9 (15%)	20 (17%)
2	2 (3%)	1 (2%)	3 (3%)
Viral pathogens^6^	13 (22%)	11 (18%)	24 (20%)
Number of viral pathogens			
0	47 (78%)	49 (82%)	96 (80%)
1	13 (22%)	10 (17%)	23 (19%)
2	0	1 (2%)	1 (1%)

^1^n = 60 children enrolled in round 1 (February 2015-February 2016 enrollment) and 60 children enrolled in round 2 (March 2016-April 2017 enrollment);

^2^Sex of the child was not able to be ascertained for 1 child;

^3^94/120 children had dates of survey and stool collection within 7 days of each other, and thus were included in analyses for diarrhea;

^4^Bacterial pathogens included in Luminex Assay: *Campylobacter* spp., *C*. *difficile*, *V*. *cholerae*, enterotoxigenic *E*. *coli* (ETEC), *E*. *coli* O157, *Salmonella enterica*, *Shigella* spp., Shiga-toxin producing *E*. *coli* (STEC), *Y*. *pestis*;

^5^Parasitic pathogens included in Luminex Assay: *Cryptosporidium* spp., *Giardia* spp., *E*. *histolytica*;

^6^Viral pathogens included in Luminex Assay: adenovirus 40/41, norovirus, rotavirus

All analyses involving reported diarrhea were limited to the 94/120 children that had surveys of reported diarrhea collected within ±7 days of the stool specimen to ensure biological plausibility of concurrence of symptoms and pathogen carriage. About 18% of caregivers reported the child had diarrhea within the past week, with half reporting having sought care for the diarrhea (Table 1b). However, among all children, 83% had ≥1 pathogen detected in their stool, and 44% had multiple pathogens detected (Table 1c). Bacterial pathogens were most commonly detected (69%), followed by viruses (20%) and parasites (19%). The most commonly detected bacterial pathogens were *Salmonella enterica* (51%), ETEC (34%), and *C*. *difficile* (22%, S1 Table). *Giardia* spp. (78%) was the most commonly detected parasitic pathogen, while norovirus (79%) was the most common viral pathogen (S1 Table).

### ARG outcomes in children

We assessed ARGs in stool specimens by group and using aggregate metrics for total genes and diversity (Table 2). Though ARGs were detected in all specimens, the most commonly detected ARGs conferred resistance to a) macrolides, lincosamides, and streptogramin b (MLS, 100%); b) tetracyclines (98%); c) β-lactams (94%); and d) aminoglycosides (84%) (Table 2a). On average, 3.1 of 5 MLS ARGs tested, 1.8 of 2 tetracycline ARGs tested, 4.6 of 55 β-lactam ARGs tested, and 1.1 of 5 aminoglycoside ARGs tested were detected in each sample. About half of children (48%) had ARGs conferring resistance to fluoroquinolones, and 38% had ARGs conferring resistance to vancomycin. ARGs conferring multidrug resistance were not common (<1%).

**Table 2:** Antimicrobial Resistance Gene (ARG) detection by resistance group in children <14 months old, Maputo, Mozambique.

	All samples	Per sample	
a) Resistance group (# ARGs in group tested)	Samples positive for ≥ 1 ARG (%)	Avg. number of positive ARGs (standard deviation)	Range of ARGs detected
Aminoglycoside (5)	101 (84.2%)	1.13 (0.67)	0–3
β-lactamase (55)	113 (94.2%)	4.58 (2.83)	0–14
Class A β-lactamase (22)	96 (80.0%)	2.74 (1.75)	0–7
Class B β-lactamase (9)	33 (27.5%)	0.29 (0.49)	0–2
Class C β-lactamase (11)	75 (62.5%)	1.24 (1.32)	0–6
Class D β-lactamase (13)	29 (24.2%)	0.31 (0.65)	0–4
Erythromycin (1)	0 (0%)	0	0
Fluoroquinolone (7)	58 (48.3%)	0.87 (1.14)	0–4
Macrolide, lincosamide, streptogramin b (5)	120 (100%)	3.09 (1.08)	1–5
Multidrug (2)	1 (0.8%)	0.02 (0.18)	0–2
Tetracycline (2)	118 (98.3%)	1.78 (0.45)	0–2
Vancomycin (2)	45 (37.5%)	0.38 (0.49)	0–1
b) Aggregate metrics	Mean (SD)	Median (Range)	
Total ARGs	12.4 (4.5)	12 (4–28)	
Shannon Index	1.6 (0.3)	1.6 (0.6–2.0)	
Inverse Simpson’s Index	4.5 (1.1)	4.5 (1.6–6.9)	

Prevalences of individual ARGs are shown in S2 Table. Of the 84 ARGs assessed per child, 9 (11%) were detected in >50% of children: 1 aminoglycoside ARG (*aadA1*); 3 β-lactamase ARGs (*SHV*, *SHV(156G)*, and *SHV(238G240E)*); 3 MLS ARGs (*ermA*, *ermB*, and *mefA*); and 2 tetracycline ARGs (*tetA* and *tetB*). Another 19 ARGs (23%) were detected in ≥10% of children: 1 aminoglycoside ARG (*aacC2*), 11 β-lactamase ARGs (*mecA*, *CTX-M-8*, *CTX-M-9*, *SHV(156D)*, *ccrA*, *ACT-5/7*, *ACT-1*, *DHA*, *LAT*, *MIR*, and *OXA-51* variants), 4 fluoroquinolone ARGs (*QnrB-1*, *QnrB-4*, *QnrB-8*, and *QnrS*), 2 MLS ARGs (*ermC* and *msrA*), and 1 vancomycin ARG (*vanC*).

On average, 12 ARGs were detected per child (range: 4–28, Table 2b). Average Shannon Index was 1.6 (range: 0.6–2), while average Inverse Simpson’s Index was 4.5 (range: 1.6–6.9).

### Unadjusted associations between demographic and enteric pathogen risk factors and ARG outcomes

We assessed associations between demographic or enteric pathogen risk factors and ARG outcomes by regression techniques with a random effect for the compound (Table 3). Stool specimens collected from children enrolled in round 2 had significantly more ARGs detected than from children enrolled in round 1 (risk ratio (RR): 1.21, 95% confidence interval (CI): 1.07, 1.38), but diversity indices did not vary significantly by round. Improved household wealth (10 percentage point change in the wealth index) was associated with fewer total ARGs (RR: 0.96, 95% CI: 0.92, 1.00). Children in households with septic tanks had moderately more ARGs detected than those in households with pit latrines (RR: 1.17, 95% CI: 1.00, 1.36).

**Table 3:** Unadjusted associations between demographic and enteric pathogen-related characteristics and ARG outcomes among children < 14 months old, Maputo, Mozambique.

Risk factor	Total ARGs RR^1^ (95% CI^2^)	Shannon Index β^3^ (95% CI^2^)	Inverse Simpson’s Index β^3^ (95% CI^2^)
Round (2 vs. 1 (ref.))	1.21 (1.07, 1.38)	-0.04 (-0.14, 0.07)	-0.06 (-0.48, 0.35)
Age (months)	1.01 (0.99, 1.04)	0.00 (-0.02, 0.02)	-0.01 (-0.0, 0.07)
Female child	0.98 (0.86, 1.09)	0.06 (-0.05, 0.17)	0.29 (-0.13, 0.71)
Child is breastfed	1.04 (0.88, 1.22)	-2.9 x 10^−3^ (-0.14, 0.13)	0.06 (-0.45, 0.57)
Wealth index^4^	0.96 (0.92, 1.00)	-0.01 (-0.05, 0.03)	-0.06 (-0.20, 0.08)
Type of sanitation			
Pit latrine	Ref.	Ref.	Ref.
Pour flush (to septic)	1.17 (1.00, 1.36)	-0.04 (-0.17, 0.09)	-0.12 (-0.65, 0.38)
Pour flush to (other)	1.05 (0.82, 1.30)	0.11 (-0.08, 0.31)	0.30 (-0.41, 1.02)
Reported diarrhea^4^	0.87 (0.71, 1.05)	0.01 (-0.13, 0.18)	-0.04 (-0.61, 0.57)
Any pathogen	1.07 (0.90, 1.25)	-0.01, (-0.14, 0.15)	-3.3 x 10^−3^ (-0.60, 0.50)
Number of pathogens	1.01 (0.94, 1.08)	9.2 x 10^−4^ (-4.9 x 10^−2^, 0.05)	0.03 (-0.16, 0.23)
Any bacterial pathogen	1.16 (1.02, 1.34)	0.04 (-0.07, 0.16)	0.21 (-0.24, 0.66)
Number of bacterial pathogens	1.10 (1.01, 1.19)	8.2 x 10^−3^ (-0.06, 0.07)	0.07 (-0.20, 0.35)
Any parasitic pathogen	0.83 (0.70, 0.96)	0.04 (-0.11, 0.18)	0.12 (-0.43, 0.67)
Number of parasitic pathogens	0.83 (0.71, 0.95)	0.03 (-0.08, 0.14)	0.08 (-0.34, 0.46)
Any viral pathogen	0.88 (0.74, 1.04)	-0.08 (-0.20, 0.06)	-0.26 (-0.75, 0.27)
Number of viral pathogens	0.93 (0.80, 1.06)	-0.06 (-0.19, 0.05)	-0.21 (-0.68, 0.34)

^1^Risk ratio estimated by mixed effects Poisson regression, with a random effect for compound;

^2^Confidence interval;

^3^Estimate is from mixed effects linear regression for the diversity metric;

^4^Limited to children whose survey for self-reported diarrhea was ≤7 days from stool specimen collection (n = 94)

Presence of ≥1 bacterial pathogen in stool was associated with 16% more ARGs detected (RR: 1.16, 95% CI: 1.02, 1.34). Each additional bacterial pathogen detected was associated with a 10% increase in ARGs (RR: 1.10, 95% CI: 1.01, 1.19). Conversely, parasitic pathogen detection was associated with 17% fewer ARGs (RR: 0.83, 95% CI: 0.70, 0.96). Pathogen detection was not associated with differences in diversity indices.

### Adjusted associations between demographic and enteric pathogen risk factors and ARG outcomes

We assessed associations between demographic or enteric pathogen carriage and ARG outcomes adjusted by round (Table 4). Adjustment for reported diarrhea for enteric pathogen risk factors—as initially designed—was instead conducted as a sensitivity analysis (S3 Table), given the 22% loss of observations from non-concurrent assessment of diarrhea and collection of stool. The wealth index was not significantly associated with total ARGs when adjusting for round (Table 4). Adjusting for round, detection of ≥1 bacterial pathogen in stool was associated with increased total ARGs (RR: 1.18, 95% CI: 1.02, 1.37), with an association of 9% more ARGs per bacterial pathogen detected (RR: 1.09, 95% CI: 1.01, 1.17). Detection of parasitic pathogens was associated with 16% fewer ARGs (RR: 0.84, 95% CI: 0.71, 0.99). Sensitivity analysis indicated no modeled estimates of enteric pathogen risk factors for total ARGs were meaningfully attenuated (maximum attenuation: 8% of the original effect estimate) when also adjusted for reported diarrhea (and therefore limited to the 94 children with surveys for reported diarrhea ≤7 days from stool specimen collection) and most were strengthened (S3 Table).

**Table 4:** Adjusted^1^ associations between demographic and enteric pathogen-related characteristics and ARG outcomes among children < 14 months old, Maputo, Mozambique.

Risk factor	Total ARGs RR^2^ (95% CI^3^)	Shannon Index β^4^ (95% CI^3^)	Inverse Simpson’s Index β^4^ (95% CI^3^)
Age (months)	1.01 (0.99, 1.03)	-3.8 x 10^−4^ (-0.02, 0.02)	4.7 x 10^−3^ (-0.08, 0.06)
Female child	0.99 (0.87, 1.11)	0.06 (-0.05, 0.16)	0.29 (-0.08, 0.74)
Child is breastfed	1.02 (0.87, 1.22)	8.6 x 10^−4^ (-0.13, 0.14)	0.07 (-0.51, 0.37)
Wealth index^5^	0.98 (0.93, 1.03)	-0.01 (-0.06, 0.02)	-0.07 (-0.23, 0.09)
Type of sanitation			
Pit latrine	Ref.	Ref.	Ref.
Pour flush (to septic)	1.04 (0.86, 1.24)	-0.02 (-0.17, 0.13)	-0.11 (-0.65, 0.55)
Pour flush (to other)	1.01 (0.79, 1.27)	0.11 (-0.06, 0.31)	0.30 (-0.39, 1.07)
Reported diarrhea^5^	0.88 (0.71, 1.08)	0.01 (-0.14, 0.16)	-0.04 (-0.66, 0.56)
Any pathogen	1.08 (0.92, 1.32)	-0.01 (-0.14, 0.13)	-0.01 (-0.60, 0.53)
Number of pathogens	1.01 (0.95, 1.08)	-4.7 x 10^−4^ (-0.06, 0.05)	0.03 (-0.17, 0.24)
Any bacterial pathogen	1.18 (1.02, 1.37)	0.03 (-0.08, 0.16)	0.21 (-0.31, 0.66)
Number of bacterial pathogens	1.09 (1.01, 1.17)	0.01 (-0.05, 0.07)	0.07 (-0.19, 0.31)
Any parasitic pathogen	0.84 (0.71, 0.99)	0.04 (-0.09, 0.17)	0.12 (-0.49, 0.67)
Number of parasitic pathogens	0.84 (0.72, 0.95)	0.03 (-0.09, 0.15)	0.08 (-0.41, 0.54)
Any viral pathogen	0.89 (0.75, 1.04)	-0.08 (-0.20, 0.05)	-0.26 (-0.83, 0.26)
Number of viral pathogens	0.93 (0.80, 1.07)	-0.07 (-0.19, 0.07)	-0.21 (-0.66, 0.27)

^1^All estimates adjusted for round;

^2^Risk ratio estimated by mixed effects Poisson regression, with a random effect for compound;

^3^Confidence interval;

^4^Estimate is from mixed effects linear regression for the diversity metric;

^5^Limited to children whose survey for self-reported diarrhea was ≤7 days from stool specimen collection (n = 94)

## Discussion

We assessed the prevalence and diversity of ARGs as our outcome, and 1) poor sanitation and other environmental risk factors; and 2) enteric pathogen carriage; in children (<14 months old) in dense, low-income compounds in Maputo, Mozambique. Total ARGs detected from stool using an 84-target array varied from 4–28 per stool specimen. The most common ARGs detected were associated with resistance to MLS, tetracyclines, β-lactams, and aminoglycosides. We did not observed significant direct associations between sanitation facilities and ARGs, though sanitation conditions were poor overall and therefore the study’s setting made it impossible to examine a wider range of WASH conditions. However, the total number of ARGs detected in a stool specimen was associated with the presence and number of bacterial pathogens detected in stool and inversely associated with presence of parasitic pathogens (mostly *Giardia* spp.), suggesting potential indirect links with WASH risk factors through enteric pathogen exposure. Differences in associations with risk factors by metric (total ARGs vs. diversity measures) may also warrant further investigation of these outcomes.

This study provides important evidence of the background carriage of ARGs in the developing gut of children in a low-income, urban environment of an LMIC and quantifies risk factors for ARG detection from the environment and concurrent enteric pathogen carriage. Although there are no studies—to our knowledge—focusing on children under 1 year of age in LMIC settings, these data are consistent with evidence from high-income settings of prevalent colonization of ARGs conferring resistance to aminoglycoside, tetracycline, or β-lactam antibiotics in the early infant gut [50–55].

In addition to the importance of ARG fate and transport in the environment and the child gut, the ARGs detected have potential clinical consequences in this setting. Most ARGs detected conferred resistance to MLS, tetracycline, β-lactam, or aminoglycoside antibiotics, with about 50% of children also having ARGs conferring resistance to fluoroquinolones. Mozambican guidelines for treatment of enteric infections in children <2 years old suggest ampicillin and gentamicin [3], which may have resistance conferred via β-lactamase [56] and aminoglycoside [57] ARGs, among others. Among children >2 years, chloramphenicol (a fluoroquinolone) is suggested [3]. Pediatric ARG surveillance is rare outside of clinical settings; however, our results indicate similarly high prevalence (≥85%) of tetracycline resistance genes (*tetA*, *tetB*) in children in this study as in previous clinical investigations of resistant *Shigella* and *Salmonella* in feces from children <5 with moderate-to-severe diarrhea in rural Mozambique [58]. Further, CTX-M gene prevalence (about 10%)—associated with extended-spectrum β-lactamase-producing *E*. *coli* and other coliforms—was lower in our study than in a recent assessment of *E*. *coli* and *Klebsiella* spp. in Mozambican university students (66%) [59]. However, given challenges in the interpretation of ARG detection as compared with phenotypic resistance [23,25], we underscore that molecular ARG detection in stool alone, even in the presence of an enteric pathogen, does not imply that the antimicrobial would not be useful for treatment of an enteric infection, nor that the pathogen is phenotypically resistant to it.

There are numerous potential sources of ARGs for the developing infant and child’s gut. Studies in high-income settings suggest infants may acquire ARGs from their mother—both with and without recent maternal antibiotic use—before, during, and just after birth, especially tetracycline and β-lactamase ARGs [43,50,51,53]. Specifically, the type of delivery (e.g. increased detection of tetracycline ARGs from the mother’s vagina in infants born vaginally vs. Cesarean section [53]), breast milk [43], and skin contact [43] may be important sources of mother-to-child ARG transmission. Longitudinal assessment of the infant gut resistome suggests environmental contributions to ARGs in the first year of life [60]. Notably, ARGs colonizing the gut may also be housed in commensal bacteria [61], suggesting both pathogenic and commensal bacterial exposures as sources.

Environmental exposure measures (e.g. type of sanitation) were not directly associated with pediatric ARG detection although emerging evidence suggests WASH facilitates the development and environmental dispersion of AR organisms at regional or national scales [5–8]. WASH conditions in study compounds were generally poor [39] and may not have improved sufficiently—even with the introduction of septic tanks—to observe or detect differences in ARGs. Given this evidence, local or site-specific sources, dynamics, and exposure pathways for AR organisms in these environments—especially with decentralized or onsite sanitation systems—are poorly understood. One study in rural Bangladeshi households with prevalent animal and human fecal contamination concluded that the physical and chemical characteristics of the environment (for example, water content of soil) were more important than household WASH characteristics in the detection of soil *E*. *coli*, many of which were pathogenic and carried clinically-relevant ARGs [62]. These findings suggest that other physical and chemical conditions of the household environment in Maputo, which we did not measure and could have changed with the introduction of water-based septic systems in round 2 in the MapSan trial [26], may also have modified WASH-AR organism relationships. Such change in environmental conditions with the introduction of septic systems may explain why significantly more ARGs were detected in round 2 children than those in round 1. Importantly, because septic systems were introduced for round 2, it was difficult to separate their associations with ARGs from the significantly higher detection of ARGs in round 2 alone, especially given small sample sizes. This could explain why septic tanks were associated with moderately higher ARG detection in unadjusted models but not in those adjusted for round.

Associations between total ARGs and carriage of enteric bacterial pathogens, including modest evidence of dose-response between total ARGs and number of bacterial pathogens, suggest these pathogens as potential vectors of ARGs and, subsuquently, indirect links with poor WASH. Data from clinical surveillance in Mozambique suggest phenotypic resistance to first- and second-line treatments for enteric infections is high among children <5 presenting with diarrhea due to enteric bacteria (e.g. *Salmonella enterica*, *Shigella* spp., ETEC [58,63]). AR in human and animal enteric pathogens is also increasing in the region [64]. Correspondingly, carriage of ARGs by enteric bacteria in the environment may concurrently increase, suggesting that continual high levels of exposure to such bacteria may transfer genotypic, and potentially phenotypic, resistance. Notably, the direction of association could also be reversed: that is, prevalent clinical treatment of pediatric diarrhea—irrespective of etiologic agent—with antibiotics and widespread non-prescription antimicrobial use in LMICs [2,3] could drive antimicrobial treatment, and subsequent selection pressure and ARG transfer. However, because we used molecular (and not culture-based) techniques and this was a cross-sectional assessment, we cannot ascertain whether the specific enteric pathogens detected in a child’s stool were also carrying the specific ARGs that were concurrently detected. Pediatric exposure to antimicrobial treatment for other infections (e.g. respiratory infections), may also be significant: in Mozambique, invasive pneumococcal disease, which affects 416/100,000 child-years, is highest in children <2 and requires treatment with penicillin and chloramphenicol [3]. Thus, concurrent antimicrobial use that we did not assess in our survey may contribute to ARG carriage.

Inverse associations between parasites—primarily *Giardia* spp.—and total ARGs may indicate that previous evidence suggesting bacterial colonization of the gut is modified with concurrent *Giardia* spp. carriage extends to ARG transfer, though this finding would be the first of its kind and should be investigated further. Although the role of *Giardia* spp. in the incidence and severity of co-infecting agents is still being studied, *in vivo* mouse studies and *in vitro* studies of human cells suggest *Giardia* spp. may modify host immune response to bacterial pathogens, including reducing inflammatory responses and shortening bacterial attachment to gut lining [65–67]. These *Giardia*-bacteria interactions may reduce the incidence of symptoms in the host, and potentially could also—directly or indirectly—play a role in reducing colonization and subsequent ARG transfer via various molecular mechanisms in the human gut [68]. Conversely, if antibiotic exposure from treatment of diarrheal infections drives ARG transfer in this environment, then the well-documented reduction in diarrhea associated with *Giardia* spp. infection [67] may also explain the reductions in ARGs detected.

Measures of gut ARG diversity were largely not associated with demographic or enteric pathogen risk factors, yet may be an important additional metric worth further study. The diversity of ARGs can provide important information about environmental exposures and changes in the gut, as in previous molecular and metagenomic analyses of gut bacteria and ARGs [43–45,69,70]. Thus, while we were not able to fully examine ARG diversity in this exploratory analysis, these metrics may warrant further assessment in future studies.

There are several limitations of this analysis. Importantly, given the absence of risk factor analyses for exposure to AR pathogens or ARGs in these settings, we used an exploratory approach that warrants caution and further investigation of significant associations detected using targeted, hypothesis-testing approaches. As noted, we were unable to quantify other potential environmental pathways of exposure to ARGs, such as food from animals treated with antimicrobials [71,72]. Although we adjusted for reported diarrhea as a proxy for recent antimicrobial treatment in a sensitivity analysis and did not observe evidence of confounding, we were unable to directly quantify the recent antibiotic or other clinical history of the child, an important potential risk factor [73]. Finally, we used a commercial array to assess pediatric AR profiles and test existing multi-target qPCR technologies for population-level ARG quantification in a low-income setting; however, the array may be more appropriate for clinically-relevant ARGs in high-income countries and therefore may not have comprised all ARGs of importance to children’s guts in LMICs. Metagenomic approaches, though costly, may be a useful alternative with enhanced capacity (through access to ARG databases) to characterize the complete molecular ‘resistome’ and identify key ARGs of public health importance with environmental transmission [70,74–76].

We observed prevalent ARGs that could confer resistance to first-line drugs for multiple infections, including enteric infections, in the guts of young children (<14 months old) in a densely-populated, low-income, urban setting. Environmental risk factors—including poor sanitation—were not directly associated with ARG detection in childrens’ guts; however, enteric bacterial pathogen carriage in stool was associated with increases in ARGs detected. This analysis provides important early data to begin elucidating the role that WASH—directly or indirectly—may play in transmission of AR in children in LMICs.

## Supporting information

S1 Table Specific enteric infections among children < 14 months old, Maputo, Mozambique. Table describing prevalence of specific enteropathogens in children’s stool.
(DOCX)

S2 Table List of ARGs and their prevalence in children < 14 months old, Maputo, Mozambique. Table describing prevalence of specific ARGs in children’s stool.
(DOCX)

S3 Table Associations between enteric pathogen-related characteristics and ARG outcomes among children < 14 months old, Maputo, Mozambique adjusted for round and reported diarrhea. Sensitivity analysis of associations between enteric pathogen detection and ARG outcomes among children with reported diarrhea outcomes.
(DOCX)
