# Supplementary material for: Gut carriage of antimicrobial resistance genes among young children in urban Maputo, Mozambique: Associations with enteric pathogen carriage and environmental risk factors
Source: PLoS One. 2019 Nov 22;14(11):e0225464. doi: 10.1371/journal.pone.0225464 (PMC6874316; doi:10.1371/journal.pone.0225464)
Supplement: S1 Table — Table describing prevalence of specific enteropathogens in children’s stool. (DOCX) [file pone.0225464.s001.docx]

S1 Table: Specific enteric infections among children < 14 months old, Maputo, Mozambique^1^

|  | | | |
| --- | --- | --- | --- |
| Enteric pathogen detected in stool specimens | Mean (standard deviation (SD)) or N (%), Round 1 | Mean (SD) or  N (%), Round 2 | Mean (SD) or  N (%), all years |
| Bacterial pathogens |  |  |  |
| *Campylobacter* spp.  *C. difficile*  *E. coli* O157  Enterotoxigenic *E. coli* (ETEC)  *Salmonella enterica*  Shiga-toxin producing *E. coli* (STEC) | 6 (10%)  6 (10%)  2 (3.3%)  17 (28%)  23 (38%)  2 (3.3%) | 7 (12%)  12 (20%)  0 (0%)  11 (18%)  19 (32%)  1 (1.7%) | 13 (11%)  18 (15%)  2 (1.7%)  28 (23%)  42 (35%)  3 (2.5%) |
| *Shigella* spp. | 2 (3.3%) | 10 (17%) | 12 (10%) |
| *V. cholerae* | 0 (0%) | 0 (0%) | 0 (0%) |
| *Y. pestis* | 0 (0%) | 0 (0%) | 0 (0%) |
|  |  |  |  |
| Parasitic pathogens |  |  |  |
| *Cryptosporidium* spp.  *E. histolytica*  *Giardia* spp. | 3 (5.0%)  1 (1.7%)  11 (18%) | 3 (5.0%)  1 (1.7%)  7 (12%) | 6 (5.0%)  2 (1.7%)  18 (15%) |
|  |  |  |  |
| Viral pathogens |  |  |  |
| Adenovirus 40/41  Norovirus  Rotavirus | 1 (1.7%)  11 (18%)  1 (1.7%) | 4 (6.7%)  8 (13%)  0 (0%) | 5 (4.2%)  19 (16%)  1 (0.8%) |

^1^n = 60 children enrolled in round 1 (February 2015-February 2016 enrollment) and 60 children enrolled in round 2 (March 2016-April 2017 enrollment)
