# Supplementary material for: Gut carriage of antimicrobial resistance genes among young children in urban Maputo, Mozambique: Associations with enteric pathogen carriage and environmental risk factors
Source: PLoS One. 2019 Nov 22;14(11):e0225464. doi: 10.1371/journal.pone.0225464 (PMC6874316; doi:10.1371/journal.pone.0225464)
Supplement: S2 Table — Table describing prevalence of specific ARGs in children’s stool. (DOCX) [file pone.0225464.s002.docx]

S2 Table: List of ARGs and their prevalence in children < 14 months old, Maputo, Mozambique

|  | | | | |
| --- | --- | --- | --- | --- |
| Type of resistance | ARG^1^  (*additional genes or groups detected from target*) | N, Rd 1 (%) | N, Rd 2 (%) | N,both rounds (%) |
| Aminoglycoside | aacC1 | 0 | 0 | 0 |
|  | aacC2 | 11 (18%) | 24 (40%) | 35 (29%) |
|  | aacC4 | 1 (2%) | 0 | 1 (2%) |
|  | aadA1 | 48 (80%) | 50 (83%) | 98 (82%) |
|  | aphA6 | 0 | 1 (2%) | 1 (1%) |
|  |  |  |  |  |
| β-lactamase |  |  |  |  |
| Class A | mecA | 31 (52%) | 28 (47%) | 59 (49%) |
|  | BES-1 | 0 | 0 | 0 |
|  | BIC-1 | 0 | 0 | 0 |
|  | CTX-M-1 type | 6 (10%) | 5 (8%) | 11 (9%) |
|  | CTX-M-8 type | 8 (13%) | 4 (7%) | 12 (10%) |
|  | CTX-M-9 type | 12 (20%) | 3 (5%) | 15 (13%) |
|  | GES  (*GES, IBC*) | 0 | 0 | 0 |
|  | IMI & NMC-A  (*NMC-A, IMI-2, IMI-3*) | 0 | 2 (3%) | 2 (2%) |
|  | KPC  (*KPC-1 – KPC-11*) | 0 | 0 | 0 |
|  | Per-1  (*Per-1, Per-3 – Per-5*) | 0 | 0 | 0 |
|  | Per-2  (*Per-2, Per-6*) | 0 | 1 (2%) | 1 (1%) |
|  | SFC-1 | 0 | 1 (2%) | 1 (1%) |
|  | SFO-1 | 0 | 0 | 0 |
|  | SHV | 41 (68%) | 43 (72%) | 84 (70%) |
|  | SHV(156D) | 11 (18%) | 14 (23%) | 25 (21%) |
|  | SHV(156G) | 40 (67%) | 44 (73%) | 84 (70%) |
|  | SHV(238G240E) | 43 (72%) | 43 (72%) | 86 (72%) |
|  | SHV(238G240K) | 0 | 1 (2%) | 1 (1%) |
|  | SHV(238S240E) | 3 (5%) | 3 (5%) | 6 (5%) |
|  | SHV(238S240K) | 1 (2%) | 0 | 1 (1%) |
|  | SME  (*SME-1 – SME-3*) | 0 | 0 | 0 |
|  | TLA-1 | 0 | 0 | 0 |
|  | VEB  (*VEB-1 – VEB-7*) | 0 | 0 | 0 |
|  |  |  |  |  |
| Class B | ccrA | 13 (22%) | 20 (33%) | 33 (28%) |
|  | IMP-1  (*IMP-1, IMP-3, IMP-4, IMP-6, IMP-10, IMP-25, IMP-26*) | 1 (2%) | 1 (2%) | 2 (2%) |
|  | IMP-12  (*IMP-12, IMP-14, IMP-16, IMP-18*) | 0 | 0 | 0 |
|  | IMP-2  (*IMP-2, IMP-8, IMP-11, IMP-19 – IMP-21, IMP-24*) | 0 | 0 | 0 |
|  | IMP-5  (*IMP-5, IMP-7, IMP-9, IMP-13, IMP-15, IMP-22*) | 0 | 0 | 0 |
|  | NDM | 0 | 0 | 0 |
|  | VIM-1  (*VIM-1 – VIM-6, VIM-8 – VIM-12, VIM-14 – VIM-20,*  *VIM-23 – VIM-26*) | 0 | 0 | 0 |
|  | VIM-13 variants | 0 | 0 | 0 |
|  | VIM-7 | 0 | 0 | 0 |
|  |  |  |  |  |
| Class C | ACC-1  (*ACC-1, ACC-2, ACC-4*) | 0 | 0 | 0 |
|  | ACC-3 | 0 | 0 | 0 |
|  | ACT-5, ACT-7 | 23 (38%) | 28 (47%) | 51 (43%) |
|  | ACT-1  (*ACT-1 – ACT-4, ACT-6*) | 5 (8%) | 8 (13%) | 13 (11%) |
|  | CFE-1 | 1 (2%) | 0 | 1 (1%) |
|  | CMY-10  (*CMY-1, CMY-8 – CMY-10, CMY-19*) | 0 | 0 | 0 |
|  | DHA  (*DHA-1 – DHA-3, DHA-5 – DHA-7*) | 13 (22%) | 15 (25%) | 28 (23%) |
|  | FOX  (*FOX-1 – FOX-7*) | 1 (2%) | 0 | 1 (1%) |
|  | LAT  (*LAT-1, LAT-3, LAT-4, CMY-2 group*) | 5 (8%) | 8 (13%) | 13 (11%) |
|  | MIR  (*MIR-1 – MIR-5*) | 14 (23%) | 25 (42%) | 39 (33%) |
|  | MOX  (*MOX-1 – MOX-7*) | 1 (2%) | 2 (3%) | 3 (3%) |
|  |  |  |  |  |
| Class D | OXA-10  (*OXA-10, OXA-11, OXA-14, OXA-16, OXA-17, OXA-19, OXA-28, OXA-35, OXA-142, OXA-145, OXA-147*) | 3 (5%) | 4 (7%) | 7 (6%) |
|  | OXA-18 | 0 | 0 | 0 |
|  | OXA-2  (*OXA-2, OXA-15, OXA-32, OXA-34, OXA-141, OXA-161*) | 6 (10%) | 5 (8%) | 11 (9%) |
|  | OXA-23  (*OXA-23, OXA-27, OXA-49, OXA-73, OXA-133, OXA-146, OXA-165 – OXA-171*) | 0 | 1 (2%) | 1 (1%) |
|  | OXA-24  (*OXA-24 – OXA-26, OXA-40, OXA-72, OXA-139, OXA-160*) | 0 | 2 (3%) | 2 (2%) |
|  | OXA-45 | 0 | 1 (2%) | 1 (1%) |
|  | OXA-48  (*OXA-48, OXA-162, OXA-163, OXA-181*) | 0 | 0 | 0 |
|  | OXA-50 variants | 1 (2%) | 0 | 1 (1%) |
|  | OXA-51 variants | 2 (3%) | 10 (17%) | 12 (10%) |
|  | OXA-54 | 0 | 0 | 0 |
|  | OXA-55  (*OXA-55, OXA-SH*) | 0 | 0 | 0 |
|  | OXA-58  (*OXA-58, OXA-96, OXA-97, OXA-164*) | 0 | 2 (3%) | 2 (2%) |
|  | OXA-60  (*OXA-60, OXA-60a – OXA-60c*) | 0 | 0 | 0 |
|  |  |  |  |  |
| Erythromycin | ereB | 0 | 0 | 0 |
|  |  |  |  |  |
| Fluoroquinolone | AAC(6)-Ib-cr | 6 (10%) | 5 (8%) | 11 (9%) |
|  | QepA  (*QepA1, QepA2*) | 0 | 0 | 0 |
|  | QnrA  (*QnrA1 – QnrA7*) | 0 | 0 | 0 |
|  | QnrB-1  (*QnrB1 – QnrB3, QnrB6, QnrB7, QnrB9, QnrB13 – QnrB18, QnrB20, QnrB23, QnrB24, QnrB29, QnrB30*) | 11 (18%) | 13 (22%) | 24 (40%) |
|  | QnrB-31  (*QnrB31, QnrB32*) | 0 | 0 | 0 |
|  | QnrB-4  (*QnrB4, QnrB11, QnrB12, QnrB22*) | 6 (10%) | 6 (10%) | 12 (10%) |
|  | QnrB-5  (*QnrB5, QnrB10, QnrB19*) | 3 (5%) | 7 (12%) | 10 (8%) |
|  | QnrB-8  (*QnrB8, QnrB21, QnrB25, QnrB27, QnrB28*) | 5 (8%) | 8 (13%) | 13 (11%) |
|  | QnrC | 0 | 0 | 0 |
|  | QnrD | 1 (2%) | 4 (7%) | 5 (4%) |
|  | QnrS  (*QnrS1 – QnrS4*) | 15 (25%) | 25 (42%) | 40 (33%) |
|  |  |  |  |  |
| MLS^2^ | ermA | 22 (37%) | 44 (73%) | 66 (55%) |
|  | ermB | 45 (75%) | 53 (88%) | 98 (82%) |
|  | ermC | 13 (22%) | 17 (28%) | 30 (25%) |
|  | mefA | 60 (100%) | 60 (100%) | 120 (100%) |
|  | msrA | 26 (43%) | 31 (52%) | 57 (48%) |
|  |  |  |  |  |
| Multidrug | orpj | 1 (2%) | 0 | 1 (1%) |
|  | oprm | 1 (2%) | 0 | 1 (1%) |
|  |  |  |  |  |
| Tetracycline | tetA | 56 (93%) | 56 (93%) | 112 (93%) |
|  | tetB | 50 (83%) | 52 (87%) | 102 (85%) |
|  |  |  |  |  |
| Vancomycin | vanB | 0 | 0 | 0 |
|  | vanC | 16 (27%) | 29 (48%) | 45 (38%) |

^1^NCBI Taxonomic ID, reproduced from: <https://www.qiagen.com/~/media/genetable/ba/antibioticresistancegenes>
