# Supplementary material for: Gut carriage of antimicrobial resistance genes among young children in urban Maputo, Mozambique: Associations with enteric pathogen carriage and environmental risk factors
Source: PLoS One. 2019 Nov 22;14(11):e0225464. doi: 10.1371/journal.pone.0225464 (PMC6874316; doi:10.1371/journal.pone.0225464)
Supplement: S3 Table — Sensitivity analysis of associations between enteric pathogen detection and ARG outcomes among children with reported diarrhea outcomes. (DOCX) [file pone.0225464.s003.docx]

S3 Table: Associations between enteric pathogen-related characteristics and ARG outcomes among children < 14 months old, Maputo, Mozambique adjusted for round and reported diarrhea (n = 94)

|  | | | |
| --- | --- | --- | --- |
| Risk factor | Total ARGs  RR^1^ (95% CI^2^) | Shannon Index  Β^3^ (95% CI^2^) | Inverse Simpson’s Index  Β^3^ (95% CI^2^) |
| Any pathogen | 1.09 (0.87, 1.37) | -0.01 (-0.20, 0.18) | 0.03 (-0.74, 0.74) |
| Number of pathogens | 1.02 (0.94, 1.11) | -0.02 (-0.04, 0.09) | 0.11 (-0.18, 0.35) |
|  |  |  |  |
| Any bacterial pathogen | 1.23 (1.03, 1.50) | 0.05 (-0.09, 0.17) | 0.27 (-0.35, 0.85) |
| Number of bacterial pathogens | 1.12 (1.02, 1.23) | 0.03 (-0.05, 0.12) | 0.15 (-0.20, 0.48) |
|  |  |  |  |
| Any parasitic pathogen | 0.85 (0.71, 1.04) | 0.07 (-0.10, 0.22) | 0.18 (-0.40, 0.78) |
| Number of parasitic pathogens | 0.83 (0.69, 0.98) | 0.07 (-0.07, 0.21) | 0.20 (-0.35, 0.74) |
|  |  |  |  |
| Any viral pathogen | 0.87 (0.73, 1.05) | -0.08 (-0.25, 0.08) | -0.25 (-0.79, 0.34) |
| Number of viral pathogens | 0.92 (0.77, 1.08) | -0.07 (-0.19, 0.07) | -0.20 (-0.74, 0.37) |
| ^1^Risk ratio estimated by mixed effects Poisson regression, with a random effect for compound; ^2^Confidence interval; ^3^Estimate is from mixed effects linear regression for the diversity metric; | | | |
